# Implementation of palliative care as a mandatory cross-disciplinary subject (QB13) at the Medical Faculty of the Heinrich-Heine-University Düsseldorf, Germany 

**DOI:** 10.3205/zma000948

**Published:** 2015-02-11

**Authors:** Christian Schulz, Ursula Wenzel-Meyburg, André Karger, Alexandra Scherg, Jürgen in der Schmitten, Thorsten Trapp, Andreas Paling, Simone Bakus, Gesa Schatte, Eva Rudolf, Ulrich Decking, Stephanie Ritz-Timme, Matthias Grünewald, Andrea Schmitz

**Affiliations:** 1 Heinrich-Heine-University, Medical Faculty, Interdisciplinary Centre for Palliative Medicine, Düsseldorf, Germany; 2 Department of Psychiatry, Harvard Medical School and Department of Psychosocial Oncology and Palliative Care, Dana Farber Cancer Institute, USA; 3 University Hospital Düsseldorf, Clinical Institute of Psychosomatic Medicine and Psychotherapy, Düsseldorf, Germany; 4 University Hospital Düsseldorf, Institute of General Medicine, Düsseldorf, Germany; 5 University Hospital Dusseldorf, Institute of Transplantation Diagnostics and Cell Therapeutics and Clinical Ethics Committee, Düsseldorf, Germany; 6 University Hospital Düsseldorf, Roman Catholic Healthcare Chaplaincy, Düsseldorf, Germany; 7 University Hospital Düsseldorf, Protestant Healthcare Chaplaincy, Düsseldorf, Germany; 8 Heinrich-Heine-University, Medical Faculty, Deanery of Student Affairs, Düsseldorf, Germany; 9 Heinrich-Heine-University, Medical Faculty, Institute of Forensic Medicine, Düsseldorf, Germany; 10 University Hospital Düsseldorf, Centre for Education and Professional Development in Healthcare, Düsseldorf, Germany; 11 Heinrich-Heine-University, Medical Faculty, Clinic of Anaesthesiology, Düsseldorf, Germany

**Keywords:** cross-disciplinary subject, QB13, palliative care curriculum, interprofessional education, virtual simulated/standardised patient contact, e-learning, group sessions for self-development and self-reflection, attitude towards palliative care

## Abstract

**Background: **By means of the revision of the Medical Licensure Act for Physicians (ÄAppO) in 2009, undergraduate palliative care education (UPCE) was incorporated as a mandatory cross sectional examination subject (QB13) in medical education in Germany. Its implementation still constitutes a major challenge for German medical faculties. There is a discrepancy between limited university resources and limited patient availabilities and high numbers of medical students. Apart from teaching theoretical knowledge and skills, palliative care education is faced with the particular challenge of imparting a professional and adequate attitude towards incurably ill and dying patients and their relatives.

**Project description: **Against this background, an evidence-based longitudinal UPCE curriculum was systematically developed following Kern’s Cycle [1] and partly implemented and evaluated by the students participating in the pilot project. Innovative teaching methods **(virtual standardised/simulated patient contacts, e-learning courses, interdisciplinary and interprofessional collaborative teaching, and group sessions for reflective self-development)** aim at teaching palliative care-related core competencies within the clinical context and on an interdisciplinary and interprofessional basis.

**Results:** After almost five years of development and evaluation, the UPCE curriculum comprises 60 teaching units and is being fully implemented and taught for the first time in the winter semester 2014/15. The previous pilot phases were successfully concluded. To date, the pilot phases (n=26), the subproject “E-learning in palliative care” (n=518) and the blended-learning elective course “Communication with dying patients” (n=12) have been successfully evaluated.

**Conclusion: **All conducted development steps and all developed programmes are available for other palliative care educators (Open Access). The integrated teaching formats and methods (video, e-learning module, interprofessional education, group sessions for reflexive self-development) and their evaluations are intended to make a contribution to an evidence-based development of palliative care curricula in Germany.

## Zusammenfassung

**Einleitung: **Im Rahmen der Novellierung der Ärztlichen Approbationsordnung (ÄAppO) im Jahr 2009 fand die Palliativmedizin als 13. Querschnittsbereich (QB 13) Eingang in die ärztliche Ausbildung als Pflichtlehr- und Prüfungsfach. Die Implementierung des neuen QB stellt nach wie vor Medizinische Fakultäten vor große Herausforderungen. Geringe Lehrressourcen und nur geringe Zahlen von Patienten stehen einer hohen Anzahl von Studierenden gegenüber. Neben der Vermittlung von Wissen und Fertigkeiten liegt in der Lehre der Palliativmedizin auch eine besondere Herausforderung in der Vermittlung einer ärztlichen Haltung gegenüber unheilbar erkrankten und sterbenden Menschen und deren Angehörigen.

**Projektbeschreibung: **Vor diesem Hintergrund wurde an der Medizinischen Fakultät der Heinrich-Heine-Universität und dem Universitätsklinikum Düsseldorf ein evidenzbasiertes longitudinales Curriculum systematisch nach dem Kern-Zyklus [1] entwickelt und teilweise bereits implementiert sowie durch die Studierenden im Pilotprojekt evaluiert. Innovative Lehrmethoden **(Virtuelle Schauspielpatienten, eLearning-Kurse, interprofessionelle Lehre und reflexive Selbstentwicklungsgruppe)** wurden mit dem Ziel eingesetzt, palliativmedizinische Kernkompetenzen interdisziplinär und interprofessionell im klinischen Kontext zu vermitteln.

**Ergebnisse: **Das gesamte in diesem Prozess entwickelte Curriculum Palliativmedizin (60 UE) wird nach einer nahezu 5-jährigen Entwicklungsphase ab dem Wintersemester 2014/2015 erstmalig in vollem Umfang durchgeführt. Die vorangestellten Pilotphasen wurden erfolgreich abgeschlossen. Bisher liegen Evaluationsergebnisse der Pilotierungsphasen (n=26), des Teilprojektes eLearning in der Palliativmedizin (n=518) und dem Blended-Learning Wahlpflichtfach „Kommunikation mit Sterbenden“ (n=12) vor.

**Schlussfolgerung: **Alle durchgeführten Schritte und entwickelten Programme stehen anderen Fakultäten zur Umsetzung frei zugänglich zur Verfügung (Open Access-Verfahren). Die eingesetzten Lehrkomponenten (Spielfilm, eLearning-Module, interprofessionelle Lehre, reflexive Selbstentwicklungsgruppe) und deren Evaluation sollen einen Beitrag zur evidenzbasierten Entwicklung palliativmedizinischer Curricula in Deutschland leisten.

## Background

In July 2009 the Lower House of the German Parliament integrated undergraduate palliative care education (UPCE) as a mandatory cross-disciplinary examination subject (QB13) into the Medical Licensure Act for Physicians when passing a bill to revise regulations regarding assistance requirements of patients with special individual needs in the hospital setting [2]. At the Medical Faculty of Heinrich-Heine-University, Düsseldorf, Germany, the resulting development of palliative care education and teaching structures coincided with a fundamental reorganisation of medical education and the development of a model study programme.

At the international level, UPCE curricula have been found to show a lack of consistency in curricular content and teaching methods, a focus on knowledge and skills rather than on the development of essential and adequate attitudes towards palliative care as well as a lack of formal assessment [3], [4]. A recent analysis of undergraduate palliative care education (UPCE) in US medical schools by Horowitz et al. highlighted a mismatch between the perceived significance and necessity of teaching palliative care competencies and the current level of palliative care instruction [5]. Two systematic literature reviews about palliative care education in the USA and the UK confirm these findings [6], [7]. The European Association for Palliative Care and the German Society for Palliative Care (EAPC and DGP) have been addressing this issue and formulated recommendations for the development of palliative care curricula [8]. The EAPC White Paper on palliative care education [9], [10] outlines 10 interdisciplinary and interprofessional core competencies relating to the most relevant principles of palliative care, advocating homogenous standards and consensually agreed norms for hospice and palliative care in Europe.

Two surveys from 2010 and 2012 investigating the current situation of palliative care education at all German medical faculties and schools confirm international findings regarding the heterogeneity of palliative care education [11], [12]. These results are of significance with regard to the curricular quality and the development of skills and competencies of medical students. A UK qualitative study conducted with newly qualified doctors in their first year of medical practice showed that medical education in palliative care had been insufficient and had not addressed the actual learning needs of the surveyed students [13]. According to a questionnaire-based study conducted with 101 medical students of two German universities, the interviewees described limited confidence regarding their knowledge base in palliative care [14]; also, more than 80% of the participating students declared feeling rather non confident or non confident in communicating the change from a curative treatment to palliative care to a patient or regarding the treatment of and care for terminally ill patients.

A major challenge of developing UPCE curricula lies in the discrepancy between high student numbers, limited available teaching resources and the clinically and ethically acceptable amount of real patient contacts [15]. Due to legal requirements, German universities were obliged to develop and implement palliative care curricula within a relatively short time. The teaching formats and methods presented in this article (video, e-learning module, interprofessional education, group sessions for reflexive self-development) are one approach to suitably meeting these challenges. The Medical Faculty of Heinrich-Heine-University, Düsseldorf, Germany, makes all its developed teaching formats available to other palliative care educators via Open Access [http://www.dfg.de/dfg_magazin/forschungspolitik_standpunkte_perspektiven/]. The present study describes the processes of developing, implementing and evaluating the UPCE curriculum of the Medical Faculty of Heinrich-Heine-University, Düsseldorf, Germany, with special reference to the integrated didactic concepts.

## Project description

### The UPCE curriculum of Heinrich-Heine-University

The teaching contents of the UPCE curriculum were integrated longitudinally into the Model Study Programme in Medicine starting in the winter semester 2013/14 with an overall amount of about 60 teaching units (1 TU=45 minutes; mandatory curriculum 39 TU, elective curriculum 21 TU) and integrating various innovative and evidence-based teaching formats.

**Figure 1:**
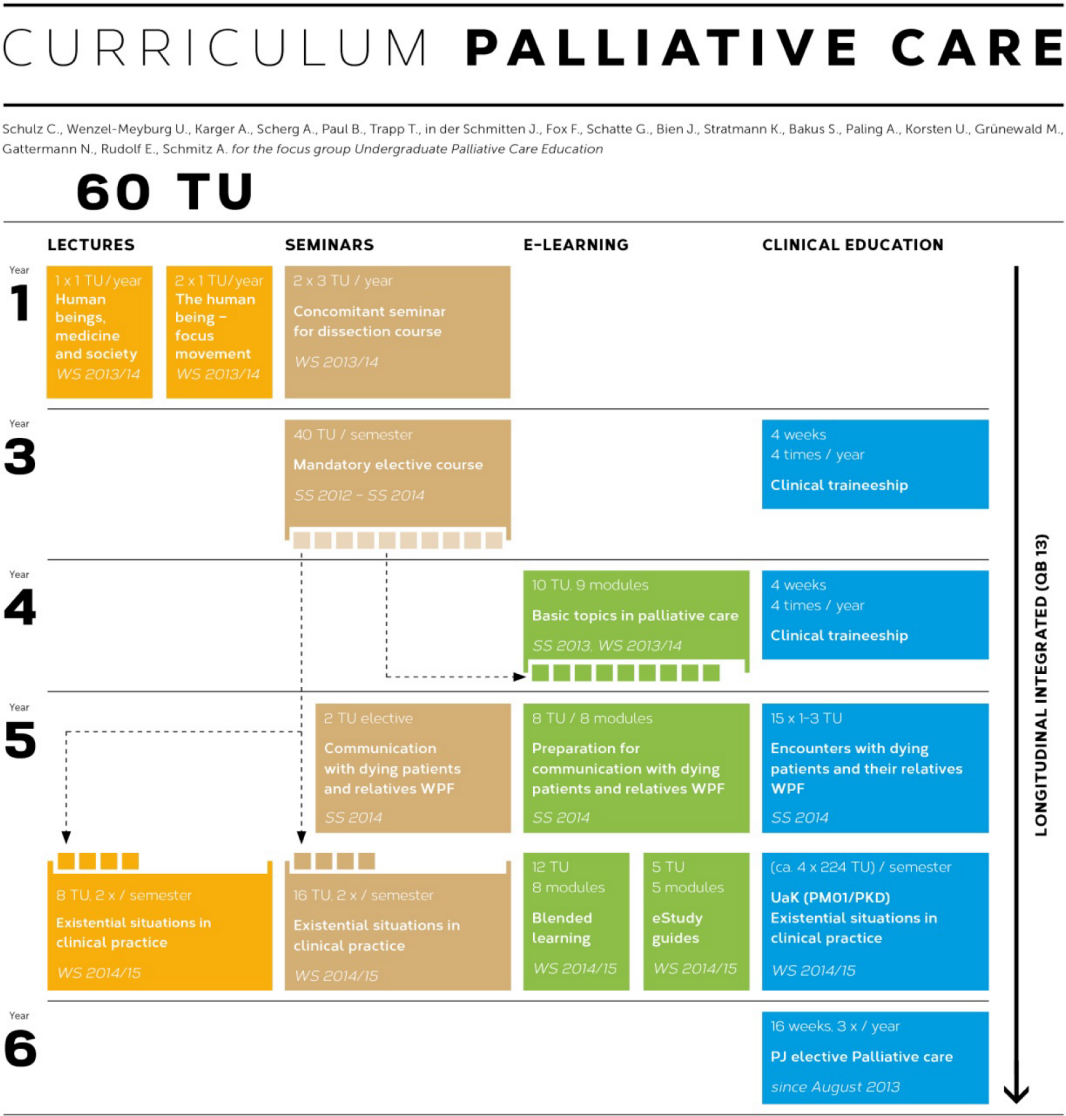
Longitudinal integration of the UPCE curriculum into the study programme in Medicine, Heinrich-Heine-University, Dusseldorf, Germany

First-year students are introduced to palliative care in the course of a lecture series offering a first presentation of various medical disciplines. In addition, the dissection course of macroscopic anatomy is supplemented by an elective **psychosocial concomitant seminar** “From the dissecting table to the sick bed”, which is taught collaboratively by the Centre of Anotomy, the Institute of Medical Sociology and the Institute of the History of Medicin as well as contributions by palliative care experts (see Figure 1 (Fig. 1)).

**Figure 2:**
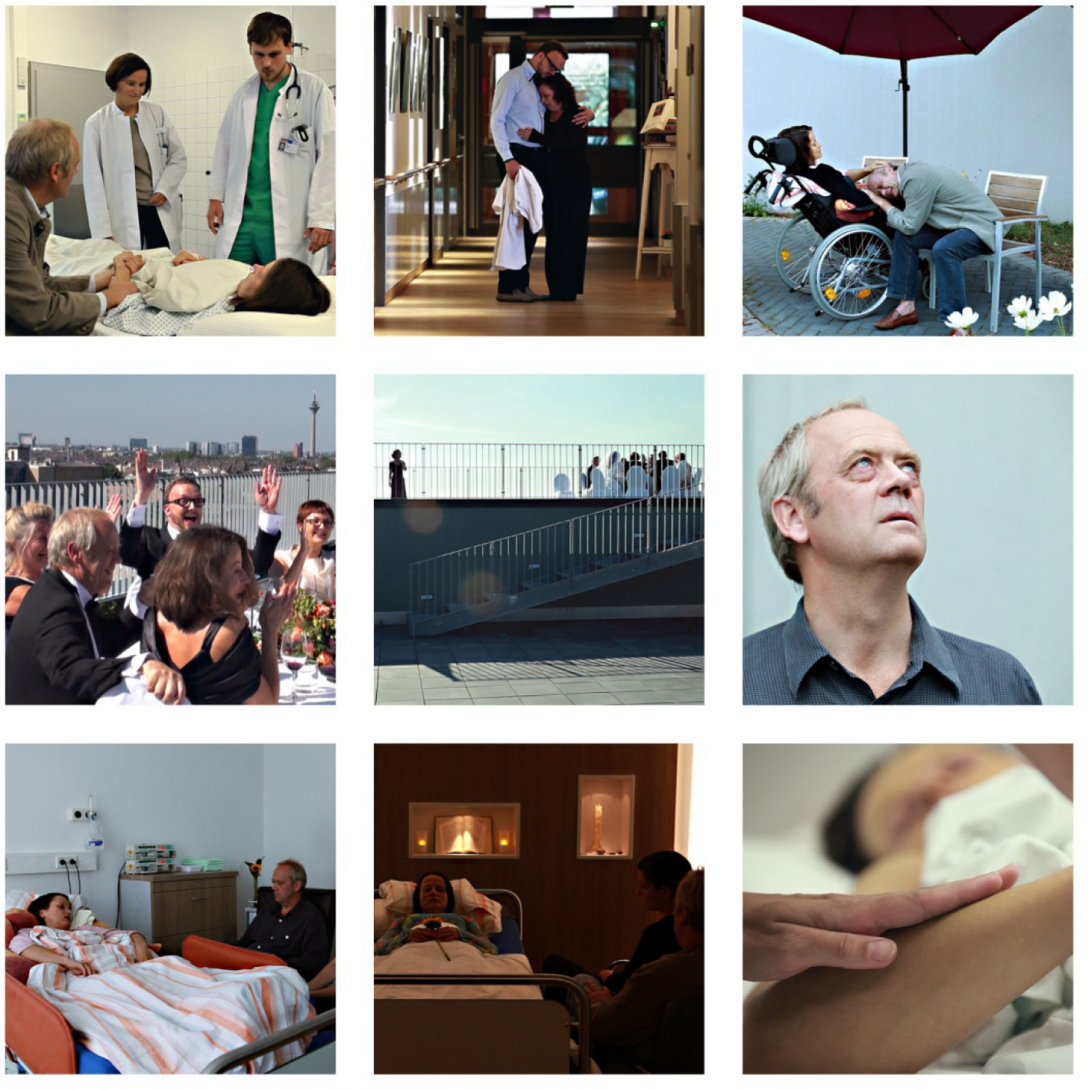
Scenes from the video „I see you“

A recurring teaching tool of the UPCE curriculum is the use of **virtual standardised/simulated patient contacts (VSP)**. VSP is based on the established didactic method of standardised/simulated patient contact (SPC) [16]. Tan et al. describe the usefulness of virtual patients to better prepare medical students for real-patient-encounters [17]. The** video “I see you”** (free download via http://mediathek.hhu.de/watch/82df7ebb-1b15-43ac-bee0-7a6e931b69dd) was produced to describe a case study example of a one-week-trajectory of a patient and her relatives on a palliative ward (see Figure 2 (Fig. 2)). The 45-minute-video aims at engendering emotional responses which will later, during the course, be used as a basis for discussion or as an introduction to various topic blocks (see Table 1 (Tab. 1)). Using VSP enables students to encounter patients with serious diseases and their relatives in a model-based environment. At the same time, model-based and distance learning provides a safe environment to approach and learn to deal with the complex issues of death and dying.

Fourth-year students have to pass an** e-learning course „Basic topics in palliative care”** consisting of 10 teaching units (TU) [18], [http://mediathek.hhu.de/watch/427905c8-00ff-46a2-9968-0a26ec0ae868]. The technical realisation of the course was effectuated via the Casus online platform®. This e-learning course is based on systematic education research and a pronounced focus on a positive and constructive learning environment (engendering affective impressions, experience of achievement, quick succession of user-repetitions) in order to effectively teach the complex issues of palliative care [19]. Apart from digital lectures, patient case vignettes, and reflective study questions with experts’ answers, the use of VSP constitutes the central teaching tool of this online course [http://www.facebook.com/pallifilm], [http://www.n-tv.de/wissen/Dem-Tod-und-dem-Sterben-ins-Auge-sehen-article12628346.html]. A test at the end of the modules prepares students for the mandatory final examination, which was held as a multiple-choice examination until the summer semester 2014, thus enabling medical students (n= 348 in the summer semester 2013 and n= 228 in the winter semester 2013/14) to obtain the mandatory examination certificate of the cross-disciplinary subject palliative care (QB13).

As of winter semester 2014/15, fifth-year students are offered the course “Existential situations in clinical practice and emergency healthcare”, which comprises 24 teaching units (see Figure 1 (Fig. 1)). As a result, the examination certificate of the cross-disciplinary subject palliative care can only be obtained upon completion of this course via a subsequent multiple-choice examination. The emphasis of the course lies in teaching discipline-specific knowledge on the basis of case-study-seminars and practical exercises of communicative encounters in palliative care via **simulated/standardised patient contact (SPC).**

Fifth-year students are also offered a mandatory elective **blended-learning course** „Communication with dying patients and relatives” [20] (see Figure 1 (Fig. 1)), which is based on an evaluated concept for undergraduate palliative care education (UPCE) [4]. At the beginning of the course, students complete eight e-learning modules (interactive learning modules, weekly chat forum) introducing them into the psychological, ethical, social and spiritual basic issues with regard to communicating with patients with advanced incurable conditions. The second phase consists of personal 1:1 encounters of students and patients. In the final third phase, students are provided the opportunity to reflect on their experiences in moderated small groups. Schulz et al. demonstrated positive effects of the course on students’ self-estimation of competence [4]. Since 2010, qualitative interviews have been published which show that patients are often willing to participate in student encounters and that they value active listening and concrete questions regarding the issues of death and dying [15].

**Figure 3:**
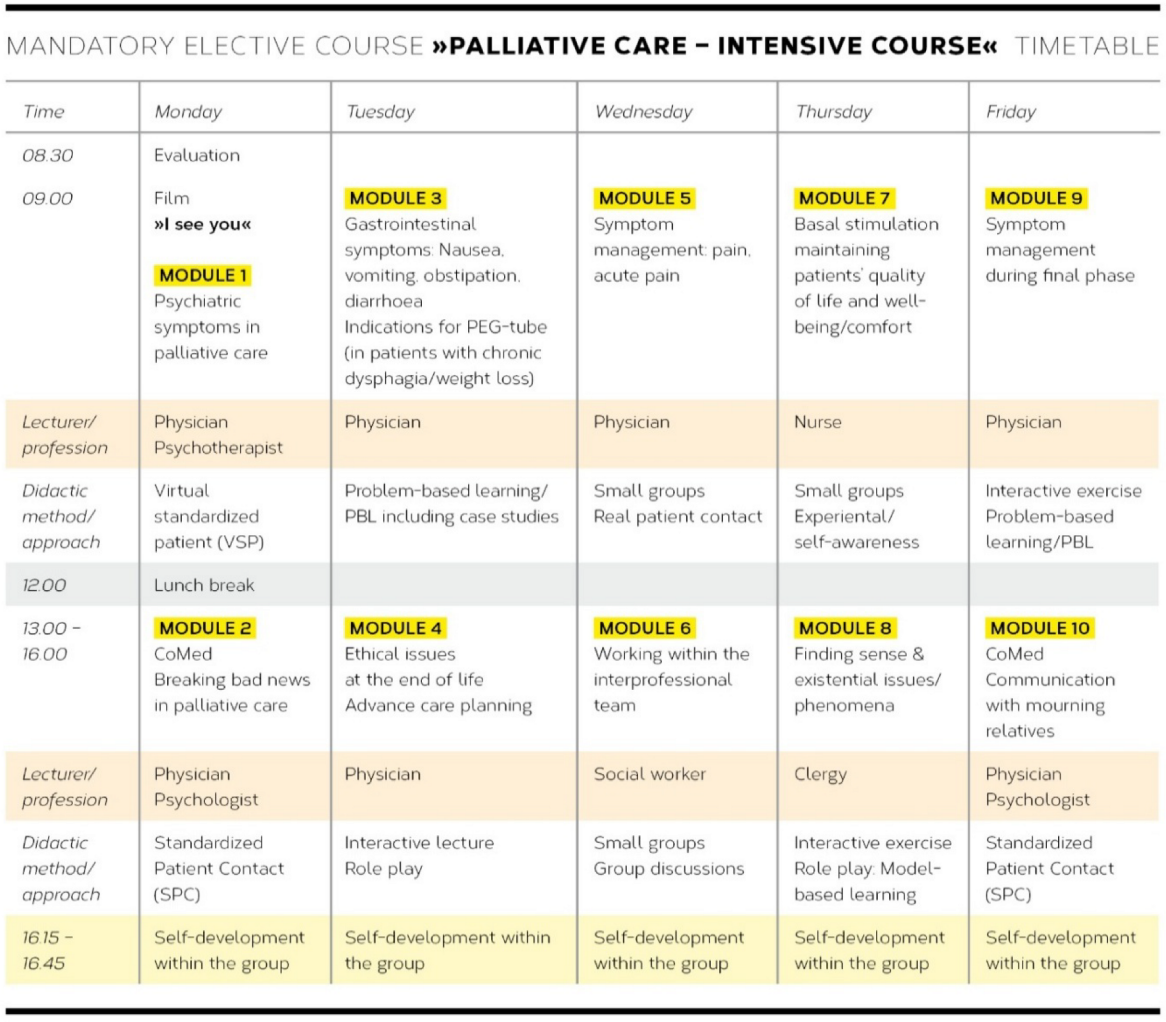
Timetable of mandatory elective course „Palliative Care – Intensive Course“ including teaching strategies

The mandatory elective course “Palliative care – intensive course” (see Figure 3 (Fig. 3)) included a daily 30-minute-**group session for reflective self-development** (see Figure 3 (Fig. 3)). This group session was moderated by a psychotherapist and aimed at enabling participating students to reflect on their attitudes on the taught topics, in particular with regard to a personal approach to death and dying, and to share emotional experiences. Research has shown that academic education rarely includes the opportunity to reflect on and discuss emotional responses [21]. It has also been demonstrated that targeted interventions dedicated to the reflection and management of strong emotional responses to palliative care experiences were regarded as helpful and necessary by the questioned participants [22], [23]. A longitudinal qualitative investigation of participants’ experiences with regard to group sessions for reflective self-development is being conducted at the moment.

In accordance with the EACP recommendations [8],** interdisciplinary and interprofessional collaborative teaching** constitutes another central didactic element of the curriculum, which was also realised by other universities in this form [24], [4]. For the healthcare professions and disciplines which are involved in this course, see Figure 3 (Fig. 3).

#### Implementation according to Kern’s approach to curricular development

**Figure 4:**
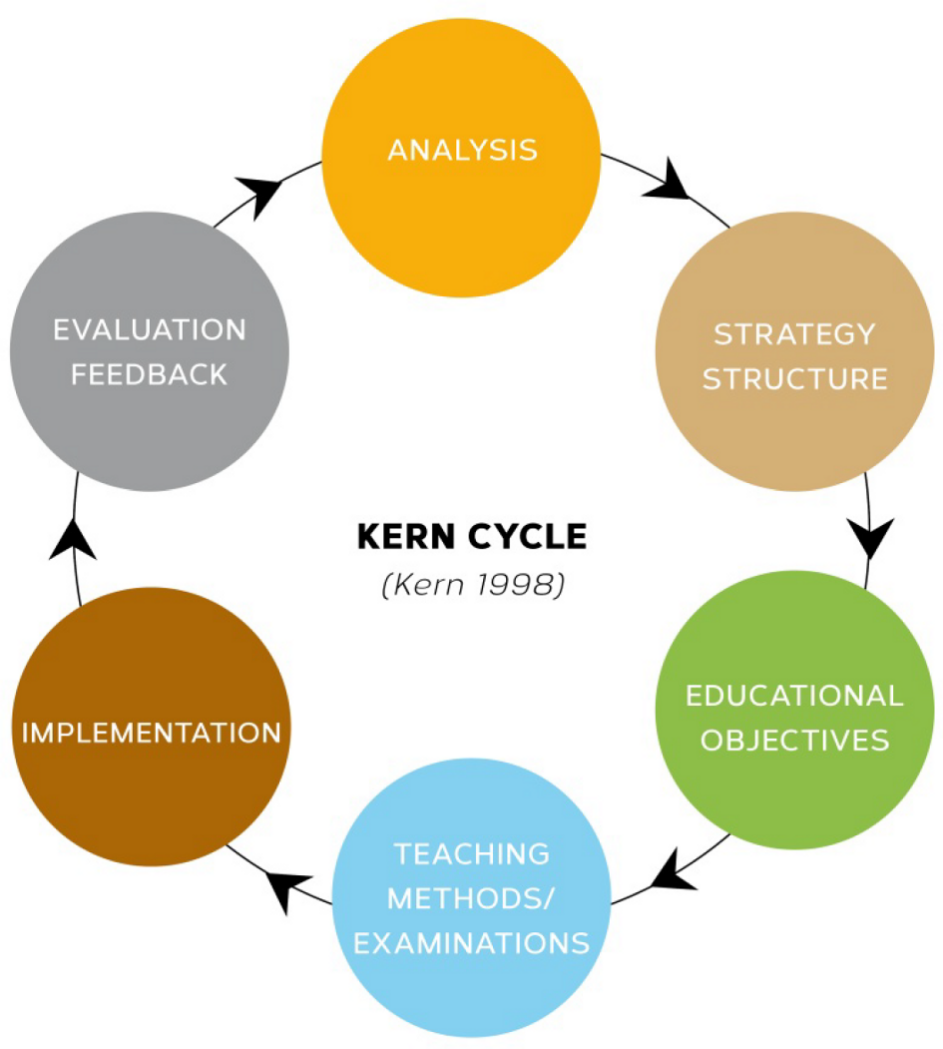
Kern Cycle – a circular approach to developing medical curricula

The application of the evidence-based framework of Kern’s approach to curricular development [1] aimed at achieving an optimal integration of various medical disciplines while avoiding curricular overload and unnecessary repetition of topics. The UPCE curriculum was developed in six steps (see Figure 4 (Fig. 4); ethics committee approval No. 4726 of the Ethics Committee of Heinrich Heine University, Germany).

The manualised UPCE curriculum was implemented in three phases. During each of the pilot phases one and two, a mandatory elective course “Palliative care – intensive seminar” consisting of 10 teaching units (TU) was developed (see Figure 3 (Fig. 3)). During phase three and following the evaluation process, nine teaching modules from the elective course were integrated into the final UPCE curriculum (see Figure 1 (Fig. 1)).

## Results

Due to a requirement analysis conducted by Heinrich-Heine-University, only selected palliative care topics had been included in its medical curriculum before the revision of the Medical Licensure Act, taught on a sporadic basis and dispersed between several different subjects (Owing to lack of space the results cannot be described in this paper). As a consequence of the new legal regulations, an interprofessional and interdisciplinary focus group to develop and foster UPCE was established (*AG Lehre Curriculum Palliativmedizin*) at the Medical Faculty of Heinrich-Heine-University and the University Hospital Düsseldorf in 2009, with quarterly meetings being held each year. Apart from the Deanery of Student Affairs of the Medical Faculty and the Interdisciplinary Centre for Palliative Medicine (IZP), the focus group consists of representatives of the following institutions: Clinic for Haematology, Oncology and Clinical Immunology, Clinic of Anaesthesiology, Institute of General Medicine, Institute of Psychosomatic Medicine and Psychotherapy, Institute of Forensic Medicine, Centre for Education and Professional Development in Healthcare, as well as representatives of the nursing profession, of medical students and the chaplaincies.

As a basis for the consecutive development steps according to Kern’s approach, an interprofessional expert panel (4 meetings) developed a catalogue of learning objectives (see Table 1 (Tab. 1)). 110 learning objectives were assigned to five contextual teaching domains, each of which also included the three subdomains knowledge, skills and attitudes: symptom management, communication and interaction, interprofessionalism, ethical/legal/social aspects, and self-reflection. (The catalogue of learning objective is available from the authors upon request.)

As of winter semester 2014/15 and after almost five years of development and evaluation, the UPCE curriculum comprises 60 teaching units and is, for the first time, being fully implemented and taught. The previous pilot phases were successfully concluded. To date, the pilot phases, the subproject “E-learning in palliative care” and the blended-learning elective course “Communication with dying patients” have been successfully evaluated. (For an overview of the UPCE curriculum, see Figure 1 (Fig. 1)).

The e-learning course “Palliative care” was evaluated by the participating students and received an overall rating of 1.9 (mean; n=300; SD=0.9) in the summer semester 2013 and an overall rating of 1.7 (mean; n=218; SD=0.7) in the winter semester 2013/14. The pilot phase mandatory elective course “Palliative care – intensive seminar” was evaluated by the participating students (n=15) on a 6-point Likert Scale with a median rating of 1.3 (range 1-2) in the winter semester 2012/13. The subsequent pilot phase mandatory elective course held in the summer semester 2013 was evaluated by the participating students (n=11) by means of the same questionnaire and received a median rating of 1.0. The qualitative student feedbacks (open answer section), in particular with regard to the e-learning course, revealed students’ requests for direct experiences with real patient contacts. The evaluation results were discussed with the UPCE focus group in an audit, which resulted in the development and implementation of the mandatory elective course “Communication with dying patients”, thus complying with students’ requests for practical work placements and real encounters with dying patients and their relatives. In the summer semester 2014, this pilot mandatory elective course was evaluated by the participating students and received an overall rating of 1.4 (mean; n=12; SD=0.5).

## Discussion

The Medical Faculty of Heinrich-Heine-University and the University Hospital Düsseldorf, Germany designed and developed a UPCE curriculum of 60 teaching units (TU) according to the Kern cycle, partly implemented it and evaluated it by means of pilot studies. The following paragraphs describe and discuss the structural, methodological, and outcome aspects of the project.

### Structural level

Various challenges affect and influence the current processes of curricular development at German medical faculties and universities. The low amount of teaching hours which are available for UPCE at most German medical faculties poses difficulties as it complicates a smooth implementation process, which particularly applies to faculties without a chair for palliative care [25]. In addition, legal requirements oblige medical faculties to implement the changes in the German Medical Licensure Act “neutrally”, i.e. without any additional credit load. Therefore the threat of escalating complexity and curricular overload in the second stage of studies is a real one because additional cross-disciplinary subjects and their examinations inevitably increase the overall study burden for enrolled students. Notwithstanding these tight schedules, according to a survey conducted by the focus group palliative care of the German Federal Association of Medical Students (bvmd) [26], medical students articulated their requests for more direct patient contact, more opportunities to consciously deal with their emotions and for learning how to adequately respond to patient needs and requests. In addition, there is a considerable divergence between infrastructural resources available at the various German medical faculties for the implementation of the new Medical Licensure Act regulations [25]. A 2012 survey highlights the faculties’ efforts to achieve a high quality of teaching with regard to the complex issues of medical treatment and care of severely and terminally ill patients. The survey also demonstrates that the amount of mandatory credit hours is higher in those faculties with an independent Chair for palliative care [11]. 

Heinrich-Heine-University, Düsseldorf, Germany, was able to combine the implementation of the new QB13 with a concurrent fundamental reform of its medical curriculum and the development and realisation of a model study programme of Medicine. This unique situation offered the opportunity to introduce innovative palliative care teaching formats and methods while optimising synergies with other medical disciplines. Regardless of these structural measures there remained a considerable discrepancy between existing resources, the available amount of reasonable real patient contacts and the overall number of students, which could only be solved by means of innovative teaching concepts and formats. As the development of this UPCE curriculum had entailed a considerable expenditure of resources with regard to time, human resources and financial costs (production of the educational video, development of the e-learning modules etc.), the UPCE focus group decided to make all teaching concepts and formats and teaching units implemented in the Düsseldorf UPCE curriculum available for any interested educational institutions (Open Acces, upon request from the authors).

#### Methodological level

According to the WHO definition of palliative care and the recommendations of the EAPC White Paper, the UPCE curriculum presented in this paper was developed by an interprofessional focus group and has been taught in interdisciplinary and interprofessional collaboration between various healthcare disciplines and professions. The evaluation of an interprofessional approach of a palliative care training programme demonstrated that the collaborative teaching by representatives of various healthcare professions was highly valued by the interviewed participants [27]. A German study by Just et al. on interprofessional education demonstrated a moderate effect of interprofessional palliative care education on the quality of interprofessional communication [28]. These results indicate that interprofessional education might positively influence the quality of patient care, for instance by improving teamwork skills [29].

The entire UPCE curriculum was designed on a module basis and is taught in teaching blocks of 90-180 minutes each, enabling a flexible use of “educational building blocks” in times of continually changing framework conditions and resources. It also provides the opportunity to implement a new curriculum in several consecutive steps, a significant advantage for faculties with a challenging environment or with a higher level of available support for successive solutions. The fact that even single and short teaching modules may have significant effects on medical students’ self-perceived knowledge and confidence as well as their attitude towards dying patients and their relatives or their interest in palliative care has been indicated by the results of a study about a ninety-minute UPCE teaching module conducted by Weber et al. [30].

A core strategy to provide adequate UPCE for high numbers of students lies in the use of e-learning and blended-learning approaches within the new curriculum. The use of e-learning modules is an engaging and suitable way to teach e.g. palliative care skills and competencies or to enable students to develop appropriate attitudes towards their future professional role [20]. The use of virtual patient case vignettes may help to solve ethical concerns about the involvement of highly vulnerable patient groups in medical education. At the same time, such a teaching format may provide a safe learning environment for approaching the difficult issues of death and dying, which may also be transferable to other educational institutions or settings [31]. It also enables educators to realistically design and edit online tools about topics and situations which do not lend themselves to traditional teaching formats (e.g. final phase, rituals following the death of a loved one, family conflicts) [18]. According to a study on student evaluations of a blended course, Kavanaugh et al. demonstrated the success of a blended-learning approach in UPCE in comparison to a traditional face-to-face teaching approach [32]. Ruiz et al. highlighted evidence for the effectiveness and acceptance of e-learning within medical education, in particular with regard to the blended-learning approach combining e-learning and traditional face-to-face teaching formats [33]. According to Kim et al. case discussions about previously completed e-learning case studies may be particularly well suited to enable students to learn from faculty how to use existing resources, how to pose critical questions and how to justify proposed solutions using evidence-based approaches [34].

Successful e-learning formats and methods necessitate adequate and reliable technical support and a suitable structural environment. It is for instance of critical importance that e-learning teaching units may be accessed and completed via various mobile devices. Such new technologies create more mobility while, on the other hand, necessitating a higher level of technical prerequisites, which may quickly lead to user-frustration without adequate support. Mobile learning (mLearning) complements traditional teaching formats in undergraduate or professional continuing education. Students or course participants may access teaching contents at any given time with their mobile devices and via learning apps or the internet, thus purposefully acquiring specific knowledge and expertise. Owing to the Open Access approach, the opportunity to learn in any given place at any given time enjoys a continually growing popularity and is also of increasing significance for the discipline of palliative care.

Meanwhile there also exist first scientific findings on the utilisation of social media in medical education [35], [36]. Neill et al. found that, during a major international emergency medicine conference, a high number of participants “tweeted” rather animatedly about clinical conference contents and materials. This high degree of social media activity was limited to a relatively small group of participants. It resulted, however, in a significantly expanded dissemination of congress contents to persons who had not been able to directly participate in the conference [34]. There is still an inexhaustible range of development potential for digital teaching formats. Therefore we expect to encounter a veritable cornucopia of novel methodological approaches and methods with regard to electronic and mobile learning in the following years.

To the knowledge of the authors of this study, the introduction of group sessions for reflective self-development (loosely based on the concept of the Balint Society) constitutes the first structured longitudinal teaching intervention integrated into a German UPCE curriculum to foster students’ self-reflection skills. It is known from the literature that it is in particular medical students’ first direct encounters with death and dying which critically influence the formation of their self-perception of professional roles [37]. The question whether medical undergraduate students indeed benefit from reflecting on their attitudes within moderated group sessions for reflective self-development is being investigated in subsequent studies. Students with a less emotionally-engaged personality showed difficulties in acquiring certain professional healthcare competencies and presented a higher resistance against changing their attitudes. A study conducted by Molinuevo & Torrubia has already demonstrated a significant influence of certain personality traits on medical student’s attitudes towards improving their communication skills [38].

#### Outcome level

The entire traditional implementation process of the UPCE curriculum is being reviewed by means of a comprehensive teaching evaluation for its effects (development of competencies and attitudes, experiential learning, sustainable learning effects). Apart from elements of standard seminar evaluation (appreciation of seminar, rating of teacher quality, total rating) this comprehensive evaluation also includes changes in medical students’ self-perceived self-efficacy and in their self-perceived competence regarding the medical treatment of and care for dying patients. First results of the pilot study using a mixed-methods approach are being published separately [39]. Whether a positive self-estimation indeed results in an improved clinical care of dying patients has not been sufficiently investigated [40]. Herein lies an important responsibility for palliative care education research [41].

After all, the quality of palliative care education has to be reflected in medical students’ measurable, long-term development of clinical competence (clinical outcome). Therefore, UPCE curricula must not only focus on theoretical knowledge and skills but must also pay particular attention to interventions regarding the development of suitable attitudes. Education research with regard to differentiating the effects of single components of the UPCE curriculum constitutes a central task for the following years. In addition, the evaluation of complex interventions like the curriculum presented in this paper should be conducted on the basis of a structured step-wise evaluation format to enable sound and methodically correct statements with regard to the efficacy of teaching concepts [4]. The question of how to assess the development of competencies by means of suitable teaching formats and methods has been discussed in the literature with particular intensity [41]. While the Medical Faculty of Heinrich-Heine-University initially chose a multiple-choice examination system for the QB13 due to limited time resources, it has been decided to supplement it by a mini-CEX-examination as a next step [41]. An evidence-based and replicable development of examination formats constitutes the basis for the comparability of assessment results of different faculties or teaching concepts [4], [6], [7]. The UPCE focus group of the German Society for Palliative Care (DGP) has developed organisational structures for this purpose.

#### Limitations

The processes of developing, implementing and evaluating a UPCE curriculum, which is presented in this paper, shows several limitations. First and foremost, it must be stated that the entire development process entailed substantial personnel and other resource expenditures. Especially the costs of the development of the new teaching concepts and the production of the educational video could not be covered by the already considerable financial support of the medical faculty and the Deanery of Student affairs alone. However, the broad and interprofessional collaboration, which had existed from the very start of this common project, has considerably contributed to its success.

The positive evaluation results of the respective pilot studies are influenced by various systemic aspects. The participants of the mandatory elective course constitute a pre-selected, non-representative sample of undergraduate students. It is to be assumed, that only those students chose this elective module who already had a high intrinsic motivation to participate. Furthermore, the pronounced contrast between the new UPCE curriculum and the traditional medical curriculum of the university might have caused a distortive effect. In the past, real patient contacts had been playing only a minor role in medical education, whereas the new model study programme places a pronounced emphasis on it. Consequently, these hitherto highly positive ratings might drop or return to normal when direct patient contact will no longer be a unique feature of palliative care education.

## Conclusion

The development of a UPCE curriculum for the QB13 is a costly and time-consuming process if it is to be based on a structured and evidence-based approach. On the other hand, the result of such a process is highly appreciated by students as well as participating patients and their relatives. E-learning, blended-learning approaches and interprofessional teaching concepts not only enable to effectively teach and manage high student numbers but also offer the opportunity to demonstrate the process of changing attitudes with regard to the adequate delivery of palliative care. The following maintenance phase will focus on sustaining the quality and continuity of the UPCE curriculum and on the assessment and evaluation of its single components by means of structured education research.

## Acknowledgements

The authors thank all students for participating in the evaluation process and Manuela Schatz for translation of the paper. The development of the UPCE curriculum was sponsored by Fund for the Development of Education and the E-Learning Fund of Heinrich-Heine-University, by students’ university fees and by a donation from the Interdisciplinary Centre for Palliative Care, University Hospital Düsseldorf, Germany.

## Competing interests

Christian Schulz is Co-Speaker of the Section Evaluation and Concomitant Research of the Focus Group Education in Palliative Care of the German Society for Palliative Care (DGP). The authors declare that there are no further competing interests.

## Figures and Tables

**Table 1:**
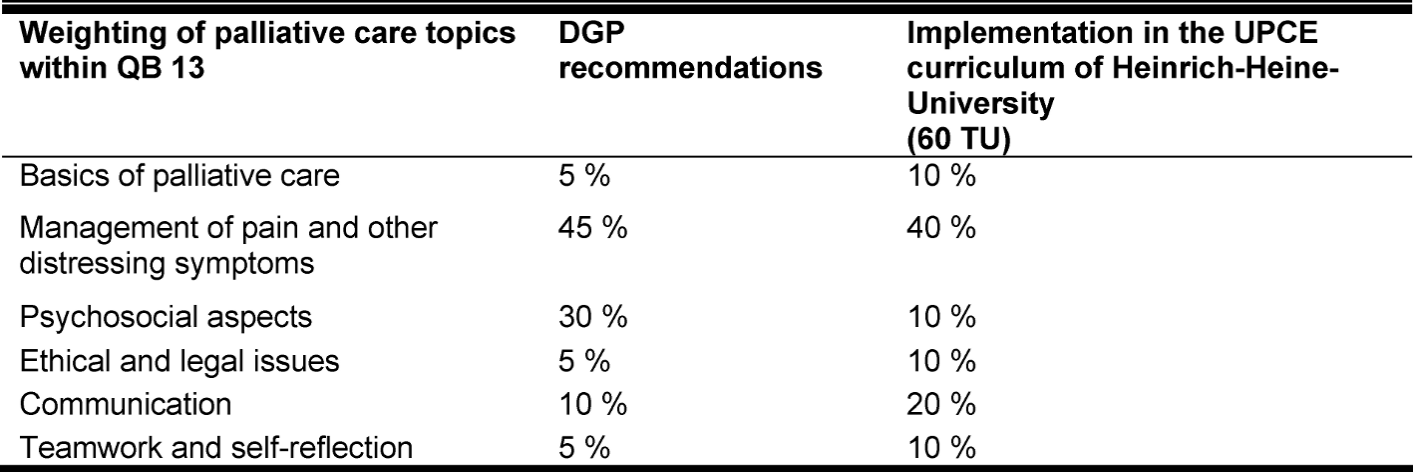
Weighting of palliative care topics within QB13 as recommended by the German Society for Palliative Care (DGP) and its implementation in the UPCE curriculum of Heinrich-Heine-University
